# Plant immunity suppression by an β-1,3-glucanase of the maize anthracnose pathogen *Colletotrichum graminicola*

**DOI:** 10.1186/s12870-024-05053-0

**Published:** 2024-04-26

**Authors:** Xiaoyu Gu, Zhiyan Cao, Zhiqiang Li, Haiyue Yu, Wende Liu

**Affiliations:** 1 College of Plant Protection, Hebei Agricultural University, Baoding, 071001 China; 2 State Key Laboratory for Biology of Plant Diseases and Insect Pests, Institute of Plant Protection, Chinese Academy of Agricultural Sciences, Beijing, 100193 China

**Keywords:** Maize, Glycosyl hydrolase, CgEC124, Callose, Immunity

## Abstract

**Background:**

Many phytopathogens secrete a large number of cell wall degrading enzymes (CWDEs) to decompose host cell walls in order to penetrate the host, obtain nutrients and accelerate colonization. There is a wide variety of CWDEs produced by plant pathogens, including glycoside hydrolases (GHs), which determine the virulence, pathogenicity, and host specificity of phytopathogens. The specific molecular mechanisms by which pathogens suppress host immunity remain obscure.

**Result:**

In this study, we found that CgEC124 encodes a glycosyl hydrolase with a signal peptide and a conserved Glyco_hydro_cc domain which belongs to glycoside hydrolase 128 family. The expression of *CgEC124* was significantly induced in the early stage of *Colletotrichum graminicola* infection, especially at 12 hpi. Furthermore, *CgEC124* positively regulated the pathogenicity, but it did not impact the vegetative growth of mycelia. Ecotopic transient expression of *CgEC124* decreased the disease resistance and callose deposition in maize. Moreover, CgEC124 exhibited the β-1,3-glucanase activity and suppresses glucan-induced ROS burst in maize leaves.

**Conclusions:**

Our results indicate that *CgEC124* is required for full virulence of *C. graminicola* but not for vegetative growth. *CgEC124* increases maize susceptibility by inhibiting host reactive oxygen species burst as well as callose deposition. Meanwhile, our data suggests that CgEC124 explores its β-1,3-glucanase activity to prevent induction of host defenses.

**Supplementary Information:**

The online version contains supplementary material available at 10.1186/s12870-024-05053-0.

## Background

Plant cell walls are the first barrier encountered by phytopathogens during the infection. Phytopathogens secrete a large number of cell-wall-degrading enzymes (CWDEs) to decompose plant cell walls in order to penetrate the host and obtain nutrients [1]. There are many types of CWDEs secreted during colonization by plant pathogens which play a critical role in the disintegration of the plant cell wall, including carbohydrate esterases (CEs), polysaccharide lyases (PLs), and glycoside hydrolases (GHs) [2]. Among them, GHs typically determine the virulence, pathogenicity, nutrient acquisition, and host specificity of phytopathogens [2–4]. For example, Ebg1, an exo-β-1,3-glucanase of the GH17 family, plays an important role in fungal cell wall integrity and virulence of the pathogen *Magnaporte oryzae*. Ebg1 can suppress β-1,3-glucan-triggered plant immunity via hydrolyzing β-1,3-glucan and laminarin into glucose [5]. It was reported that the entire *Ustilago maydis* endo-xylanase family which are comprised by two enzyme Xyn1 and Xyn2 from GH10, one enzyme Xyn11A from GH11, and one enzyme Xyn3 from GH43. All these endo-xylanases except Xyn3 are secreted and play different roles in the infection process [6]. Moreover, Erc1 (enzyme required for cell-to-cell extension), a conserved effector, is required for virulence of the corn smut *U. maydis* in maize leaves. Erc1 contributes to suppress β-glucan-induced plant immune responses by binding to host cell wall components and exhibiting 1,3-β-glucanase activity [7]. Besides, XEG1, a *Phytophthora sojae* glycoside hydrolase family 12 protein, act as a dominating virulence factor during soybean infection. Interestingly, XEG1 not only exhibit xyloglucanase and β-glucanase activity but can also induce plant defense responses via acting as a PAMP (pathogen-associated molecular pattern) in soybean [8]. These results indicate that GHs represent an important fungal strategy in the competition between phytopathogens and plants. In addition to degrading cell walls, CWDEs also act as effectors, inhibiting or eliciting plant immunity [9, 10]. During infection, the rice blast fungus *M. oryzae* secretes a glycoside hydrolase MoCDIP4, which targets the OsDjA9-OsDRP1E protein complex and inhibits rice immunity [11]. PsXEG1, and enzyme secreted by *P. sojae* enters the plant exosomes and is recognized by the Leucine-rich repeat (LRR) receptor protein XEG1 to activate the defense responses [12, 13]. Whereas, the elicitor activity of PsXEG1 does not depend on its xyglucanase activity [8].

In general, a variety of virulence effectors are secreted into the plant apoplast during the biotrophic phase of biotrophic and hemibiotrophic pathogens [14, 15]. To defend against pathogens, plants have developed a sophisticated immune system to recognize pathogen-associated molecules and activate defense responses. Plant immune responses typically include the activation of mitogen-activated protein kinase (MAPK) signaling, callose accumulation, reactive oxygen species (ROS) burst, pathogenesis-related gene expression and hypersensitive response (HR) [15, 16]. Amplified immune responses typically induce disease resistance and the accumulation of defense molecules that plants utilize to defend themselves against pathogen invasion [17].

It is well established that callose plays as an important role as a component of the plant cell wall, affecting various processes of plant development and responses to a variety of abiotic and biotic stresses [18]. Upon biotic or abiotic stresses, callose quickly accumulates in plant cell walls, plasmodesmata, and pollen cell wall [19–21]. Especially, callose can be abundant in plasmodesmata and different amount of callose deposition can regulate the movement of some signaling molecules from cell-to-cell, including RNA, metabolites and hormones [22]. In addition to its specific function in plant growth and reproduction, callose plays an important role in plant immunity [23]. It has been shown that a conserved *AtUBAC2*, which encodes homologs of UBIQUITIN-associated domain-containing protein 2, could regulate pathogen-induced callose accumulation in plant immunity [24]. It is well known that callose in the form of an amorphous, high molecular weight polysaccharide β-1, 3-glucan, which is widely found in the cell walls of many higher plants, acts as a substrate to deposit antimicrobial compounds, thereby providing a concentrated chemical defense at the site of the cell attack [23]. It has been demonstrated and generally accepted that callose as glucans can be degraded by β-1, 3-glucanases [25], and this process is a part of plant basal defense responses [25, 26].

*C. graminicola* is the causal agent of anthracnose stalk rot (ASR) and leaf blight, one of the most common and consistently damaging disease in maize [27]. ASR can reduce maize yield via disrupting the translocation of carbohydrates, photosynthesis and causing plant death [28]. Most plant pathogens can be classified into necrotrophic or biotrophic based on their feeding strategies. However, *C. graminicola* use a multistage hemibiotrophic infection strategy that appears to be an intermediate between necrotrophic and biotrophic. First, a dome-shaped appressoria puncture the host surface via a combination of mechanical forces and enzymatic degradation. Next, the bulbous biotrophic hyphae are enveloped by host plasma membrane to develop inside living cells in order to obtain nutrients from living cells. Finally, the fungal switches to a necrotrophic phase and the rapidly growing hyphae then kill and destroy host tissues [28].

In this study, we investigated a *C. graminicola* effector with a typical predicted signal peptide and a conserved Glyco_hydro_cc domain which belongs to the glycoside hydrolase 128 family, named as *CgEC124* (*C. graminicola* effector 124). We found that CgEC124 homologous proteins were highly conserved in many fungal species. The functionality of the predicted signal peptide of CgEC124was verified via the yeast signal trap method and TTC assay. *CgEC124* expression pattern was investigated using qRT-PCR and we found that the expression of *CgEC124* gene was induced in the early stage of *C. graminicola* infection, indicating that *CgEC124* may play an important role in *C. graminicola* pathogenicity. *CgEC124* gene knockout revealed that *CgEC124* is required for virulence of *C. graminicola*, but not for vegetative growth and ecotopic transient expression of *CgEC124* in maize plants confirmed the hypothesis that *CgEC124* positively contribute to *C. graminicola* pathogenicity.

Moreover, CgEC124 could inhibit callose content and exhibit the β-1,3-glucanase activity to suppress glucan-induced ROS burst in maize leaves. Taken together, *CgEC124* might play a critical role during the infection of *C. graminicola* and utilizes its β-1,3-glucanase activity for preventing induction of host defenses.

## Result

### Identification of *CgEC124* in *C. graminicola*

A previous study predicted 1650 secreted protein genes in *C. graminicola*, among them five gene categories relevant to pathogenicity were identified, containing transcription factors, secondary metabolism enzymes, candidate secreted effectors (CSEPs), carbohydrate-active enzymes (CAZymes), and transporters, which showed dramatically different expression patterns during infection [28]. Among them, a total of 327 CAZymes belong to 66 glycoside hydrolase family. We are interested in the new glycoside hydrolase 128 family, which has been reported that ThGhd_7, a member of the GH128 family in *Tilletia horrida*, was found to be able to regulate plant immunity by blocking the production of reactive oxygen species [29]. Since the research on GH128 family which modulated maize immunity has not been reported, we found that there are only two members of the GH128 family in *C. graminicola*, GLRG_08167 and GLRG_08872. Based on the phenotypes of knockout mutants and pathogenicity identification, we finally selected GLRG_08167 for the further study. A candidate effector protein named GLRG_08167, encodes a 325 amino acid protein with a typical signal peptide and a conserved Glyco_hydro_cc domain belonging to the glycoside hydrolase 128 family (Figure S1A). In this study, this effector was named *C. graminicola* effector candidate 124 (CgEC124). To identify the homologous genes in other fungi and construct a phylogenetic tree, the CgEC124 amino acid sequence was used for a Blastp search. Multiple sequence alignment and phylogenetic tree analyses revealed that CgEC124 homologous proteins were highly conserved in these fungal species (Figure S1B). Moreover, these highly homologous proteins share a common conserved Glyco_hydro_cc domain with CgEC124 (Figure S1B).

### Expression of the *CgEC124* gene was induced during infection

**Fig. 1:**
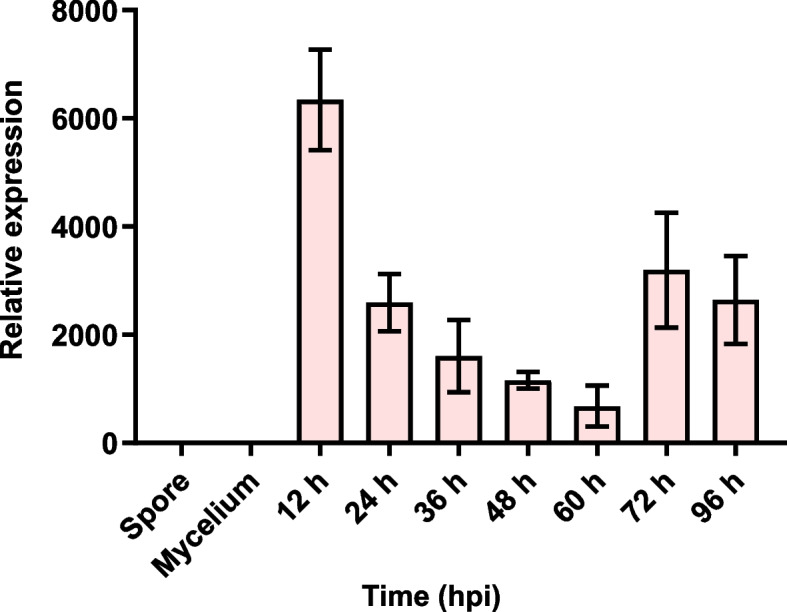
Expression pattern of *CgEC124* at different stages. Samples were taken from vegetative stages (spore and mycelium) in wild-type strain Tz-3 and invasive stages (12, 24, 36, 48, 72, and 96 hpi) in maize leaves. Expression pattern analysis was performed using qRT-PCR and *CgActin* was used as the reference gene. The bar represents the standard deviation of three biological replicates

To further investigate the function of CgEC124, we investigated the gene expression pattern of *CgEC124* using qRT-PCR in vegetative stages (spore and mycelium) and invasion stages (12 h, 24 h, 36 h, 48 h, 60 h, 72 h, and 96 h post-inoculation) of maize plants inoculated with *C. graminicola*. The expression level of *CgEC124* was barely detected in the spore and mycelium stages, while the expression level of *CgEC124* was significantly induced at the initial infection stage (12 hpi) and kept a relative high expression level at the subsequent infection stage compared with the vegetative stages (Fig. 1). These results revealed that CgEC124 may play an important role in participating in the pathogenicity of *C. graminicola*.

### CgEC124 is a secreted protein in *C. graminicola*

**Fig. 2:**
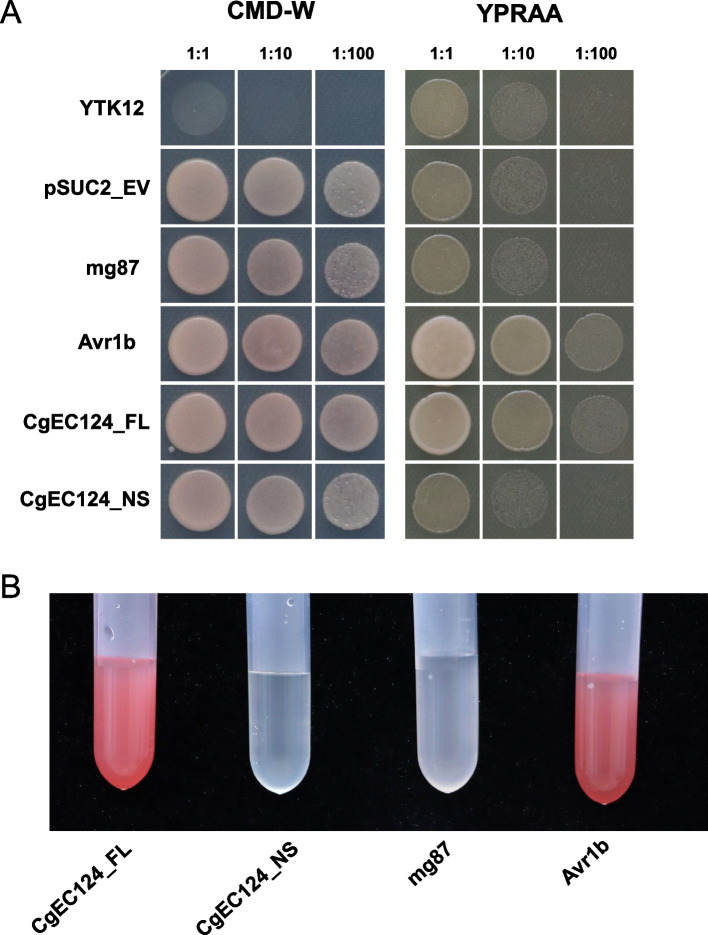
Functional validation of the CgEC124 signal peptide. **A** The secretion of CgEC124 signal peptide was verified by yeast signal trap assay. The full-length sequence of CgEC124_FL and the no signal peptide sequence of CgEC124_NS were fused into pSUC2 vector and transformed into yeast strain YTK12, respectively. Avr1b was used as the positive control, and mg87 was used as the negative control. **B** TTC assay of CgEC124 signal peptide. All experiment were repeated three times with similar results

**Fig. 3:**
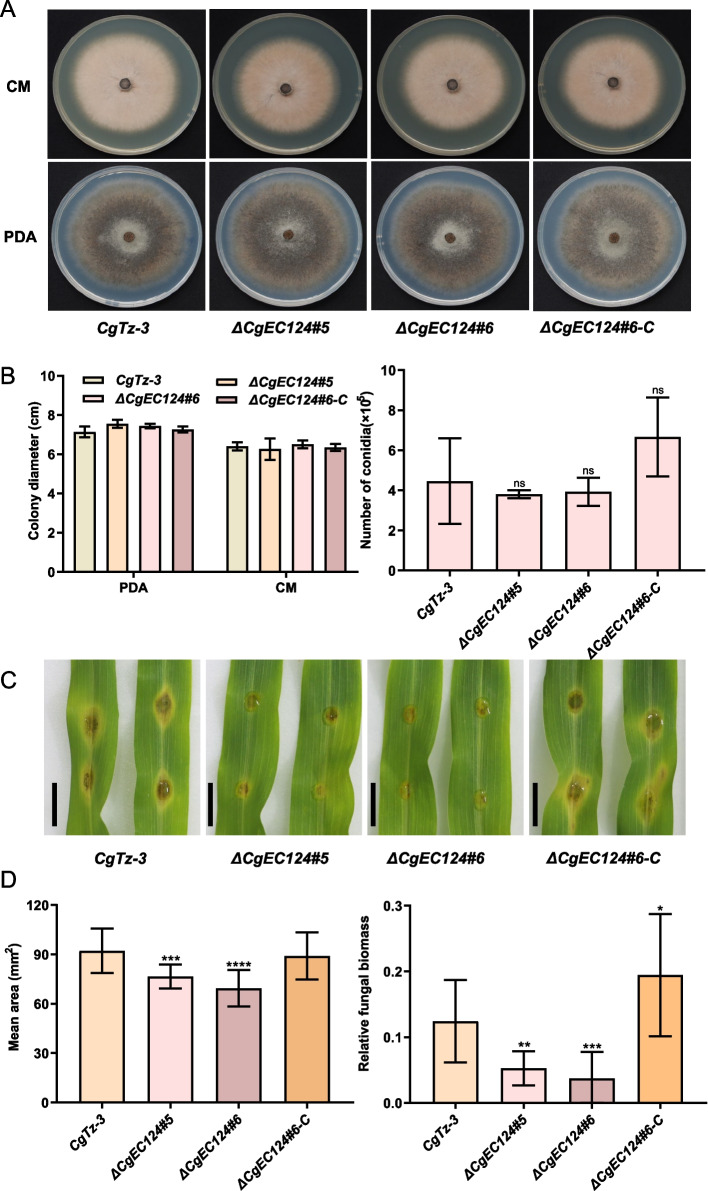
CgEC124 is required for the pathogenesis of *C.graminicola*. **A** Colony morphology of CgTz-3, *ΔCgEC124#5* and *ΔCgEC124#6*, and complementation strain *ΔCgEC124#6-C* grown on CM and PDA medium. **B** Statistical analysis of the colony diameters and conidiation. Bar indicates the standard deviation of three replicates. Significance analysis was analyzed by Student’s *t*-test and ns means no significance. **C** Pathogenicity verification of *ΔCgEC124* mutants and complementation strain in maize detached leaves. Photos were taken after 5 days post inoculation (dpi). **D** Statistical analysis of the lesion area and relative fungal biomass. Statistical significance was determined with Student’s *t*-test, ^*^p < 0.05, ^**^p < 0.01, ^***^p < 0.001, ^****^p < 0.0001

The candidate effector CgEC124 possesses a signal peptide predicted by SignalP5.0. We used the yeast signal trap method to verify the function of the predicted signal peptide. The full-length sequence (FL) and the fragment without signal peptide sequence (NS) of CgEC124 were fused into the vector pSUC2 and then transformed into the yeast strain YTK12. As shown in Fig. 3, the YTK12 containing pSUC2-CgEC124_FL, pSUC2-CgEC124_NS, pSUC2-Avr1b, and pSUC2-mg87 exhibited normal growth in the CMD-W medium. By contrast, only YTK12 containing pSUC2-CgEC124_FL, and pSUC2-Avr1b displayed normal growth in the YPRAA screen medium containing raffinose as the only carbon source (Fig. 2A). Moreover, the TTC assay was also performed to further confirm the functionality of the CgEC124 predicted signal peptide. The results showed that the YTK12 containing pSUC2-CgEC124_FL, and pSUC2-Avr1b could turn 0.1% TTC into the insoluble red-colored triphenylformazan, whereas the negative control 0.1% TTC solution remained colorless (Fig. 2B). These results verified the secretion function of the predicted signal peptide of CgEC124.

### CgEC124 is required for full virulence of *C. graminicola*

To further investigate the biological function of *CgEC124*, we constructed the *CgEC124* gene knockout mutants using the homologous recombination method and confirmed it by PCR (Figure S2A-C). We randomly selected two of the *ΔCgEC124* mutant strains *ΔCgEC124#5* and *ΔCgEC124#6* for further investigation. Moreover, *ΔCgEC124#6* was chosen to generate the gene complementation strains, which were also confirmed by PCR (Figure S2D). The *ΔCgEC124* mutant strains and complementation strains exhibited no significant phenotypic changes both on CM and PDA plates compared with the wild-type strain CgTz-3 in terms of colony diameter and conidiation (Fig. 3A, B). However, the pathogenicity assay demonstrated the reduction in virulence of *ΔCgEC124#5* and *ΔCgEC124#6* mutant strains compared to the wild type strain CgTz-3 after 5 days inoculation in detached maize leaves, whereas the complementation strains recovered the defective virulence of *ΔCgEC124* mutants (Fig. 3C, D). Moreover, the results of DAB staining showed that the ROS accumulation in FoMV:CgEC124-VOX plants was apparently less than in FoMV:GFP-VOX plants (Figure S4). These results indicated that CgEC124 is required for virulence of *C. graminicola* but not for vegetative growth.

**Fig. 4:**
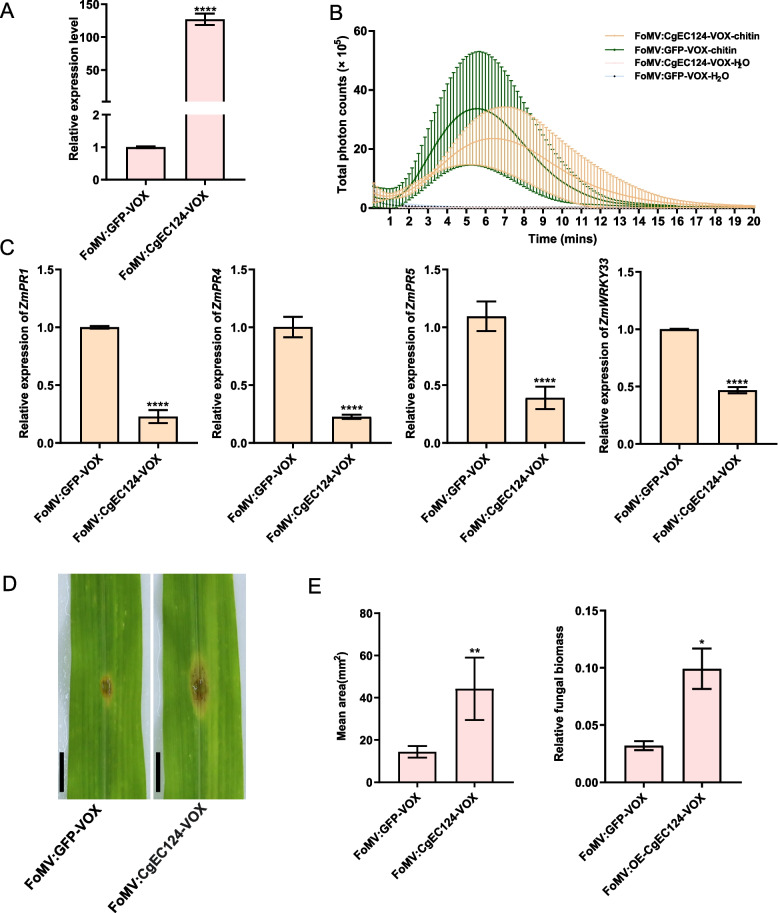
Ecotopic transient expression of *CgEC124* decreased the disease resistance in maize. **A** Transcript level of *CgEC124* in FoMV:GFP-VOX and FoMV:CgEC124-VOX plants. Bar indicates the standard deviation of three replicates. **B** Chitin-induced ROS burst in FoMV:GFP-VOX and FoMV:CgEC124-VOX plants. Bar indicates the standard deviation of three replicates. **C** The expression levels of *ZmPR1*, *ZmPR4*, *ZmPR5*, and *ZmWRKY33*, in FoMV:GFP-VOX and FoMV:CgEC124-VOX plants. Statistical significance was determined with Student’s *t*-test, ^****^p < 0.0001. **D** Phenotype of FoMV:GFP-VOX and FoMV:CgEC124-VOX plants inoculated with WT strain CgTz-3. Scare bars represented 1 cm. **E** Statistical analysis of the lesion area and relative fungal biomass. Statistical significance was determined with Student’s *t*-test, ^*^p < 0.05, ^**^p < 0.01

Furthermore, we ecotopic transiently expressed *CgEC124* in maize plants via virus-induced overexpression (VOX) method to explore whether *CgEC124* could decrease plant resistance to *C. graminicola* [30]. The FoMV-inoculated maize plants exhibited mosaic symptoms at 7 days post-inoculation (dpi) (Figure S3). We found that the relative expression level of *CgEC124* in the transient overexpression plants was highly induced compared to the FoMV:GFP-VOX plants (Fig. 4A). We further detected chitin-induced ROS burst in FoMV:CgEC124-VOX and FoMV:GFP-VOX plants. The results showed a reduced ROS burst in FoMV:CgEC124-VOX plants compared to FoMV:GFP-VOX plants (Fig. 4B). Complementary to the ROS burst assay in leaf disks in FoMV:CgEC124-VOX and FoMV:GFP-VOX plants, qRT-PCR was performed to analyze the expression levels of PR genes in ecotopic transient expressed *CgEC124* plants. The results revealed a significant decrease in the expression levels of *ZmPR1*, *ZmPR3*, *ZmPR5*, and *ZmWRKY33* genes in FoMV:CgEC124-VOX plants compared to FoMV:GFP-VOX plants (Fig. 4C). After inoculation with CgTz-3, FoMV:CgEC124-VOX plants showed increased susceptibility compared to FoMV:GFP-VOX plants (Fig. 4D, E), indicating CgEC124 positively contribute to pathogen pathogenicity.

### CgEC124 inhibited callose deposition and exhibited β-1,3-glucanase activity to suppress glucan-induced ROS burst in maize leaves

**Fig. 5:**
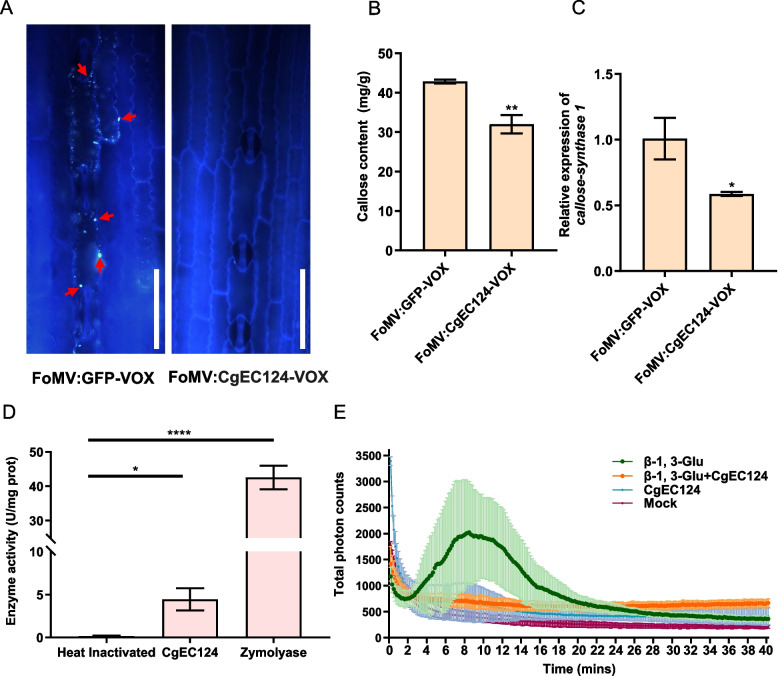
CgEC124 prevents induction of host defenses. **A** Aniline blue staining in FoMV:GFP-VOX and FoMV:CgEC124-VOX maize leaves. Aniline signals were taken at 2 h after aniline treatment. Scare bars = 50 μm. Red arrows indicate callose in the plant cell wall. Similar results were observed in three independent biological experiments. **B** Callose content in FoMV:GFP-VOX and FoMV:CgEC124-VOX plants. Statistical significance was determined with Student’s *t*-test, ^**^p < 0.01. **C** The expression level of *Callose-synthase 1* in FoMV:GFP-VOX and FoMV:CgEC124-VOX plants. Statistical significance was determined with Student’s *t*-test, ^*^p < 0.05. **D** The hydrolytic activity on β-glucan of recombinant CgEC124 glucanase. Zymolyase, a known β − 1,3-glucanase served as a positive control, and the heated inactivated CgEC124 as negative control. Statistical significance was determined with Student’s *t*-test, ^*^p < 0.05, ^****^p < 0.0001. **E** A ROS-burst assay was performed with maize leaf disks following incubation with β-D-glucan, CgEC124, and a mix of β-D-glucan and CgEC124 recombinant proteins. Bar indicates the standard deviation of three replicates

It was previously reported that callose can accumulate at the infection site to prevent fungal penetration [31]. To validate whether CgEC124 could interfere in callose deposition, we analyzed the callose content and observed the callose deposition using aniline blue staining in FoMV:CgEC124-VOX and FoMV:GFP-VOX plants. The results showed that the intensity of aniline blue signal and the callose content were significantly decreased in FoMV:CgEC124-VOX plants compared with FoMV:GFP-VOX plants (Fig. 5A, B). To further confirm that the callose content in FoMV:CgEC124-VOX and FoMV:GFP-VOX plants, we measured the expression level of *callose synthesis gene 1* (*ZmCAS1*) using qRT-PCR. The result showed a significant decrease in the expression levels of *ZmCAS1* in FoMV:CgEC124-VOX plants compared to FoMV:GFP-VOX plants (Fig. 5C). Since CgEC124 was predicted to be a member of glycoside hydrolase 128 family and have β-1, 3-glucanase activity. We further investigate its enzymatic activity functions during *C. graminicola* infection by fusing CgEC124 with a 6 × His tag and expressing it in *Pichia pastoris* (Figure S5). As expected, CgEC124 displayed β-1,3-glucanase activity (Fig. 5D). The heated inactivated CgEC124 was treated as negative control and a known β − 1,3-glucanase Zymolyase was used as positive control.

It has been shown that β-1,3-glucan can be recognized by plant cells to induce defense responses, including ROS bursts [32]. To explore whether CgEC124 affected plant defenses, induced by β-1, 3-glucan, the β-1, 3-glucan-induced ROS burst assay was performed on maize leaf disks. β-1, 3-glucan alone induced ROS burst in the maize leaf disks whereas the burst was significantly reduced by addition of CgEC124 protein. Moreover, only CgEC124 protein itself could not induce a significantly ROS burst (Fig. 5E). This indicates that CgEC124 is required for suppression of the β-1, 3-glucan-induced ROS burst. Taken together, these data suggest that CgEC124 could prevent induction of host defenses.

## Discussion

Pathogen effectors can interfere with host immunity and subvert plant defenses [33]. In this study, we characterized the effector CgEC124 and showed that is involved in *C. graminicola* pathogenicity and can decrease disease resistance by preventing induction of host defenses. CgEC124 contains an N-terminal signal peptide for extracellular secretion and a conserved Glyco_hydro_cc domain which belongs to glycoside hydrolase (GH) 128 family (Figure S1). CgEC124 plays a key role in the pathogenicity of *C. graminicola*. However, knockout of *CgEC124* does not affect its growth phenotype (Fig. 3A, B), which was observed in many studies on effectors in other pathosystems. For example, a plant peroxisome-targeting effector MoPtep1 is required for the virulence of *M. oryzae* but did not affect the fungal vegetative growth [34]. The effector gene *PsAver3c* from the soil-borne root rot pathogen *Phytophthora sojae* is required for full virulence of *P. sojae* and the *PsAvr3c* knock-out mutation did not exhibit any growth changes compared with the WT strain [35]. Our results also demonstrate that effectors may play a key role as pathogenic factors in fungal vegetative growth.

Plant pathogens possess a wide range of cell wall degrading enzymes (CWDEs) including GH family which have been reported to play an important role in penetrating plant cell wall and are generally considered to proceed necrotrophy stage, thus killing the host to acquire nutrients [36, 37]. However, the expression level of *CgEC124* was significantly induced at the initial infection stage (12 hpi), representing biotrophic growth (Fig. 1). The limited number of CWDEs at biotrophy stage can prevent the induction of host immunity via either acquiring nutrients or sequestering PAMP molecules [3]. *Histoplasma* Eng1 has evolved a specialized pathogenesis function to remove exposed-glucans, thereby enhancing the ability of yeasts to escape detection by host phagocytes [38]. The conserved effector Erc1 of corn smut *U. maydis* can bind to host cell wall components and hydrolyze β-1, 3-glucan to attenuate β-glucan-induced defense responses [7]. Moreover, MaChia1 is a chitinase from M. *oryzae* that can bind and sequester fungal-released chitin, thus preventing chitin-induced immune response [15]. In addition to the increased expression level of *CgEC124* at the initial infection stage, we found that there was also an induction at necrotrophy stage. We hypothesize that CgEC124, apart from its role as a glycoside hydrolase involved in the degradation of plant cell walls, may also enter the plant host and interact with potential targets to further regulate plant disease resistance. Therefore, future work should investigate the possible host candidate targets and explore the molecular mechanism of its inhibition of plant immunity.

*CgEC124* plays a positive role in contributing to pathogen pathogenicity (Fig. 3C). In the transient overexpressed *CgEC124* plants, a reduced ROS burst was observed compared to control plants (Fig. 4B). The reduction in PR genes expression in *CgEC124*-VOX plants suggests that *CgEC124* act upstream of these immune-related genes (Fig. 4C). We hypothesize that overexpression of CgEC124 subsequently decreased the activation of PR protein, leading to a reduced ROS burst. In addition, CgEC124 accepts β-1, 3-glucan as substrate and exhibits β-1, 3-glucanase activity to inhibit β-1, 3-glucan-induced ROS burst (Fig. 5D, E). However, CgEC124 itself is not PAMP, CgEC124 protein could not induce a significantly ROS burst in maize leaf disks. In some cases, some GH family proteins or their released products can be recognized as PAMPs. For example, a GH12 β-glucanase XEG1 from the soybean pathogen *P. sojae*, a GH45 cellulase (EG1) from *Rhizoctonia solani*, and a secreted xyloglucanase BcXYG1 from *Botrytis cinerea* are recognized as PAMPs, triggering plant cell death and immune responses [8, 39, 40]. The digesting products released by *M. oryzae*-secreted proteins MoCel12A and MoCel12B (β-1, 3–1,4- glucanase) from rice cell wall and *Cladosporium fulvum* CfGH17-1 (β-1, 3-glucanase) from tomato cell wall serve as DAMPs which active plant immune responses during infection [1, 41].

A variety of plant species such as wheat, barley, rice, and sorghum contain β-1,3-linked glucan in plant cell walls [42]. In addition, plants can strengthen their cell wall with papillae that be composed of β-1, 3-glucan-rich callose at sites of filamentous fungi penetrations [43]. The β-1, 3-glucan fragment of plant origin is almost indistinguishable from that of microbial origin that are well-described as elicitors to activate plant immune responses, such as callose (another β-1, 3-glucan) deposition at infection sites [7, 32, 44]. In previous report, callose is described as another β-1, 3-glucan can accumulate at the infection site to prevent fungal penetration [31]. Chitosan-induced callose deposition is apparent after two hours of treatment in *Arabidopsis* [21]. In our study, the intensity of aniline blue signal and the callose content were significantly decreased in *CgEC124* transient overexpression plants compared to GFP plants (Fig. 5A, B). Since callose act as a β-1, 3-glucan and CgEC124 was predicted to belong to be a member of glycoside hydrolase 128 family and have β-1, 3-glucanase activity. We hypothesize that overexpression of CgEC124 subsequently degraded the callose, leading to a decreased callose content. The expression level of *callose synthesis gene 1* (*ZmCAS1*) confirmed these results (Fig. 5C). In this regard, we hypothesize that CgEC124 prevents the accumulation and subsequent recognition of these DAMP molecules by hydrolyzing β-1, 3-glucan at the biotrophic stage. This is also consistent with the observation that CgEC124 significantly inhibited β-1, 3-glucan-induced ROS burst (Fig. 5E) and suppressed *PR* gene expression (Fig. 4C).

## Conclusions

In summary, the secreted effector CgEC124 is a member of the glycoside hydrolase 128 family with a conserved Glyco_hydro_cc domain. The expression level of *CgEC124* was induced during the *C. graminicola* infection especially in the early stage of infection. *CgEC124* is required for full virulence of *C. graminicola* and its knockout decreased the disease resistance by inhibiting host reactive oxygen species bursts as well as callose content. Lastly, CgEC124 may utilize its β-1,3-glucanase activity and suppress glucan-induced ROS burst in maize leaves.

## Materials and methods

### Fungal strains and Plant growth conditions

The *C. graminicola* strain Tz-3 was used as the wild type and the other specific knockout mutant strains were obtained based on it. All the *C. graminicola* strains were cultured on complete medium (CM) agar plates, potato dextrose agar (PDA), and oatmeal agar (OA) plates at room temperature (25℃) for 2 weeks. The *C. graminicola* strain plates were cultivated under light for spore accumulation and collection. The maize B73 inbred line was used as the wild-type (WT) plant in this research. Maize seeds were sown in pots containing a soil mixture of nutrient soil and vermiculite (3:1) and were cultivated in the greenhouse at 25 ℃ under a 16-h:8-h day:night photoperiod.

### Bioinformatic and phylogenetic analysis of CgEC124 homologous proteins

The CgEC124 amino acid sequence of *C. graminicola* and other homologous proteins were downloaded from the Ensemble Fungi database (http://fungi.ensembl.org/index.html). The phylogenetic tree was constructed by using MEGA11 software with the neighbor-joining method. The functional domain of CgEC124 was predicted by the Simple Modular Architecture Research Tool (SMART, http://smart.embl-heidelberg.de/). The Glyco_hydro_cc domain was constructed by the software DOG 2.0 [45].

### Yeast secretion trap assay

The signal peptide of CgEC124 was predicted by SignalP-5.0 [46]. In brief, the full length sequence of CgEC124 (CgEC124_FL) and no signal peptide sequence of CgEC124 (CgEC124_NS) were fused into the pSUC2 vector and transformed into the yeast strain YTK12 [47]. These recombinant strains were cultured on the yeast minimal medium with sucrose (CMD-W medium: 0.67% yeast nitrogen base without amino acids, 0.074%-Trp DO supplement, 2% sucrose, 0.1% glucose, 1.5% agar) and YPRAA medium (1% yeast extract, 2% tryptone, 2% raffinose, and 1.5% agar with 2 µg/mL antimycin A) to detect invertase secretion. The recombinant strain Avr1b (pSUC2-Avr1b) was used as a positive control and mg87 (pSUC2-mg87) was used as a negative control. In addition, the secretion characterization of CgEC124 was confirmed by the reduction of TTC (2,3,5-triphenyltetrazolium chloride, T8170, Solarbio) to detect the insoluble red triphenylformazan, as described by Yin et al. [48].

### CgEC124 gene expression analysis

To detect the expression level of CgEC124 at different stages, we collected the samples containing conidia, hyphae, and maize leaves after being inoculated by *C. graminicola*. The conidia were collected from 15-day OA plates and vegetative hyphae were harvested from 100 mL liquid CM medium after 36 h of cultivation. The maize leaves were sprayed with conidial suspensions at the concentration of 1 × 10^5^ per ml and the second leaves were collected at 12 h, 24 h, 36 h, 48 h, 60 h, 72 h, and 96 hpi. Total RNA was extracted using a FOREGENEPlant Total RNA Isolation Kit according to the manufacturer’s instructions. The first-strand cDNA was synthesized using a HiScript II 1st Strand cDNA Synthesis Kit (Vazyme, R212-01). qRT-PCR was performed using a RealStar Fast SYBR qPCR Mix (GeneStar, A301-01) with 7500 real-time PCR system (Applied Biosystems). The *CgActin* was used as the internal reference gene and the primers were listed in Table S1. The relative expression levels were calculated via 2^−ΔΔCt^ method with three technical repeats.

### Construction of *CgEC124* knockout and complementation strains

*CgEC124* gene knockout strains were constructed via the homologous recombination strategy and identified by PCR method as described previously [49]. Briefly, the hygromycin B (400,052-20 mL, MERCK) phosphotransferase gene was performed to replace the target gene for constructing the knockout mutants. For the complementary construction, the open reading frame (ORF) sequence of CgEC124 with1.5 kb native promoter region was amplified and inserted into PGTN vector and then transformed into *ΔCgEC124* protoplasts. The G418 (G6021-5 g, MACKLIN) resistance was used for screening the transformants. All the strains were identified by PCR method and primers were listed in Table S1.

### Pathogenicity analysis

To investigate the pathogenicity of different *C. graminicola* strains, 12 day-old maize seedlings were inoculated with *C. graminicola* conidial suspensions at a concentration of 1 × 10^5^ conidia per ml in 0.01% Tween-20. 10 μL of conidia droplets was dropped on the second detached maize leaves for inoculation. The infected maize leaves were cultivated with 95% humidity at 25℃ overnight and then incubated at a 14-h/10-h light /dark photoperiod. Photographs were taken at 5 days after inoculation. The lesion area was calculated using ImageJ software. The relative fungal biomass was tested via qPCR with specific primers listed in Table S1.

### Generation of ecotopic transient expression of *CgEC124* in plants

Agrobacterium-mediated ecotopic transient expression of *CgEC124* in maize plants were performed as described previously [30, 50]. Briefly, the coding sequence of *CgEC124* without signal peptide was cloned into FoMV-pCAMBIA1380 binary vector and transformed into *Agrobacterium* GV3101. The cells were harvested and resuspended in infiltration buffer (10 mM MgSO_4_, 200 μM acetosyringone) to an OD600 of 1.0. The suspension was injected into 2–3 mm above the coleoptilar node of 5-day old seedlings and cultivated for 14 days to observe the mosaic symptoms. The expression level of *CgEC124* in FoMV:CgEC124-VOX plants was detected by RT-qPCR. The fourth to sixth maize leaves were collected and lyophilized. Next, rub-inoculation was used to generate a large number of maize seedlings for the following inoculation assay.

### Heterologous protein expression in *Pichia pastoris*

The *pichia* expression system was performed for Myc-His tagged CgEC124 recombinant protein as described previously [1]. The *pichia pastoris X-33* was used as the host strain and pPICZαA vector was used for secreted protein expression. We cloned the coding sequence of CgEC124 into pPICZαA and introduced the plasmid into *pichia pastoris X-33* strain using the electroporation method. Next, *X-33* strain with corresponding constructs were cultured in BMGY (buffered glycerol–complex medium) for 1 d. Then the culture was transferred to BMMY (buffered methanol–complex medium) and added a final concentration of 0.5% methanol for expression and induction for 1 d. Recombinant proteins were harvested and concentrated. Subsequently, the protein was stored at − 80 °C for further experiments. Western blot analysis was performed to detect the target protein with the antibody anti-His (ABclonal, Cat. #AE003, dilution 1:5000).

### Enzyme activity assay

To determine the enzymatic activity of the purified CgEC124 protein, the β-1,3-glucanase (β-1,3-GA; Spectrophotometer) assay kit (Solarbio, Cat, #BC0360) was used to explore whether the recombinant protein has β-1,3-glucanase activity. Briefly, 1 µg of CgEC124 protein from *pichia pastoris* was diluted in 100 µL to incubate with 100 µL laminarin in 37℃ water bath for 1 h. Then 600 µL of 3, 5-dinitrosalicylic acid was added into the mixed solution and incubated in boiled water bath for 5 min and cooled down until room temperature. The absorption was recorded using spectrophotometer (Macy, China) at 540 nm. Here, zymolyase [38] (MK, Cat, #MF0210) and heat-inactivated recombinant protein were used as positive and negative controls, respectively.

### ROS burst assay

For ROS burst assay induced by β-1,3-glucan, maize leaf disks were incubated with distilled water in a 90 mm plate overnight in the dark. Three leaf disks were taken into a 100 µl reaction mixture which containing a luminol solution (100 µM L-012 and 20 µg ml^−1^ HRP). The following reaction mixtures were used for the ROS burst assay: (1) Buffer 20 mM KPO4 (pH 6.0) (2) 250 µM β-1, 3-glucan, (3) 3 µM CgEC124, (4) 250 µM β-1, 3-glucan incubated with 3 µM CgEC124. The luminescence was collected using a Glomax20/20 luminometer (Promega) with the following settings in a reader: Kinetic cycle: 60, interval time: 1 min.

For the ROS burst induced by chitin, the FoMV:GFP-VOX and FoMV:CgEC124-VOX maize leaves were used. Three maize leaf disks were transferred to a 1.5 mL tube with 100 μL luminol reaction buffer (Bio-Rad Immun-Star horseradish peroxidase substrate), 1 μl of horseradish peroxidase (HRP), and 1 μl of sterile water or 1 μl of 0.8 mM chitin.

### DAB staining

For DAB staining, the maize leaves were completely immersed in a 1 mg/mL DAB solution and the solution was vacuum infiltrated for 30 min. Then the leaves were incubated on a shaker for 18 h in 80 rpm and were kept in the dark. After 18 h, the leaves were moved into 90% ethanol until the chlorophyll was completely removed and then the samples were photographed.

### Callose content analysis in maize leaves

The callose content was detected using a Callose Assay Kit (Suzhou Comin Biotechnology company, Fluorescence method, 100 tubes/96 samples, www.cominbio.com) according to the manufacturer’s instructions. Callose was extracted in maize leaves and measured with multimode reader (SKL-0701). The callose reacted with aniline blue, which can produce a fluorescent substance with fluorescence intensity at excitation wavelength 400 nm and emission wavelength 500 nm.

### Aniline blue staining

For aniline blue staining, the maize leaves were bleached using ethanol until the samples were transparent. Next, 150 mM K_2_HPO_4_ was vacuum infiltrated in the leaves to replace the extra ethanol three times every 10 min. After removing the buffer, samples were covered with 0.01% aniline blue solution (w/v in 150 mM K_2_HPO_4_) at room temperature and maintained in the dark for 2 h. Confocal microscopy (Leica, Germany) was carried out with DAPI channel (Emission 490–520 nm).

### Supplementary Information

**Supplementary Material 1.**

## Data Availability

The CgEC124 amino acid sequence of C. graminicola and other homologous proteins were downloaded from the Ensemble Fungi database (http://fungi.ensembl.org/index.html). The raw data presented in the article and Supplementary materials are available from the corresponding author on reasonable request.
