# Supplementary material for: Plant immunity suppression by an β-1,3-glucanase of the maize anthracnose pathogen Colletotrichum graminicola
Source: BMC Plant Biol. 2024 Apr 26;24:339. doi: 10.1186/s12870-024-05053-0 (PMC11046878; doi:10.1186/s12870-024-05053-0)
Supplement: Supplementary file 1 — Supplementary Material 1. [file 12870_2024_5053_MOESM1_ESM.zip › SUPPLEMENTARY FIGURE 1.pdf]

SUPPLEMENTARY FIGURE 1

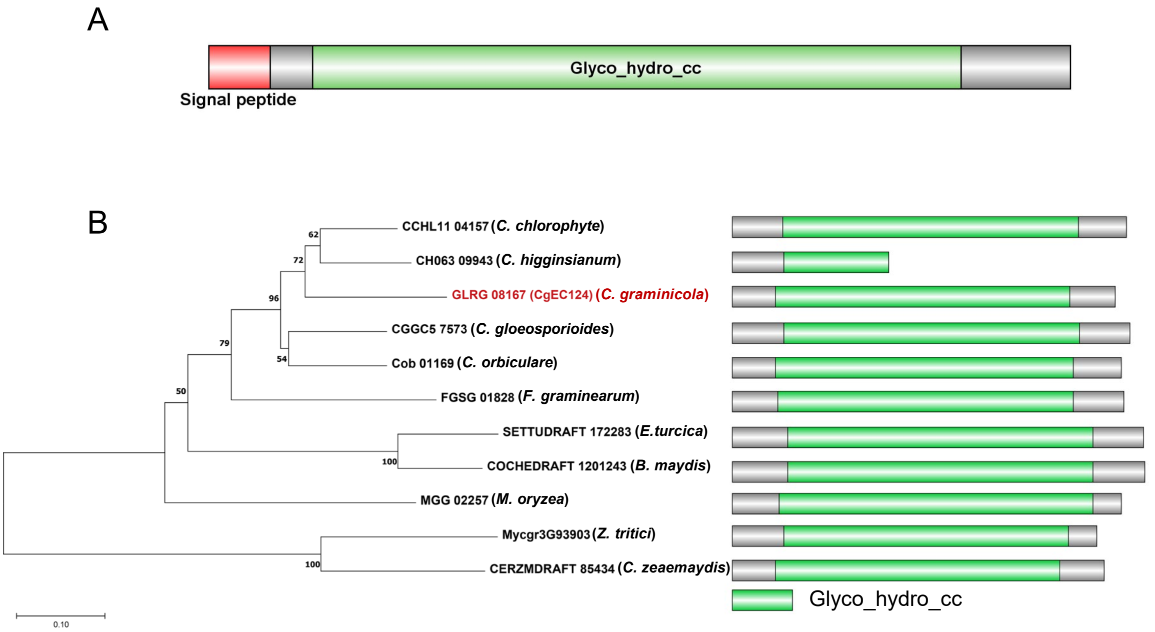

SUPPLEMENTARY FIGURE 1 Identification of CgEC124 in *C.graminicola*. (A) Functional domain analysis of CgEC124. The signal peptide was predicted by SignalP-5.0. (B) Phylogenetic relationship and domain analysis of CgEC124 and its homologs. Phylogenetic tree was constructed by the neighbour-joining (NJ) method with 1000 bootstrap values in MEGA7 and was optimized with the online tool iTOL. The scale bars represent a genetic distance of 0.01 substitutions per nucleotide position.
