# Supplementary material for: Plant immunity suppression by an β-1,3-glucanase of the maize anthracnose pathogen Colletotrichum graminicola
Source: BMC Plant Biol. 2024 Apr 26;24:339. doi: 10.1186/s12870-024-05053-0 (PMC11046878; doi:10.1186/s12870-024-05053-0)
Supplement: Supplementary file 1 — Supplementary Material 1. [file 12870_2024_5053_MOESM1_ESM.zip › SUPPLEMENTARY FIGURE 2.pdf]

SUPPLEMENTARY FIGURE 2

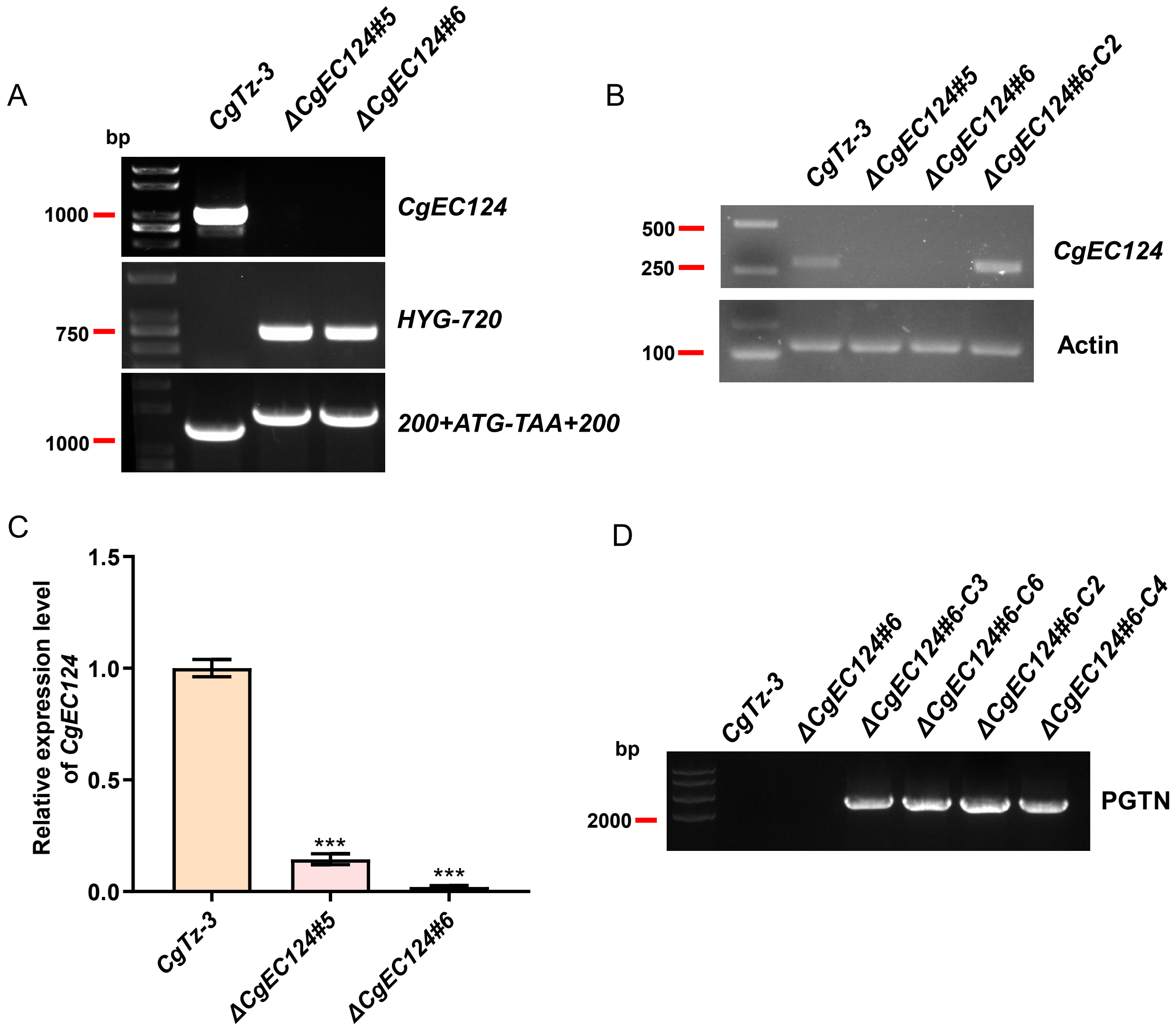

SUPPLEMENTARY FIGURE 2 Construction and identification of *CgEC124* transformants using PCR methods. (A) Identification of the Δ*CgEC124* knockout strains. The wild-type strain Tz-3 and two independent *ΔCgEC124* mutant strains were analyzed via PCR method with three pairs of primers. *CgEC124* gene fragments could not be detected in *ΔCgEC124* mutant strains, hygromycin specific primer HYG-700 could be amplified in *ΔCgEC124* mutant strains, and ATG+300/TAG+300 fragments showed different length in Tz-3 and *ΔCgEC124* mutants. (B) Semiquantitative PCR validation of the different strains. cDNA of the four indicated strains above were used as template. *CgEC124* gene fragments could not be detected in *ΔCgEC124* mutant strains, but can be detected in WT and complementary strain. (C) Identification of the *ΔCgEC124* mutant strains using qRT-PCR method. *CgEC124* gene expression level was hardly detected in *ΔCgEC124* mutant strains. (D) Identification of the complementary strain Δ*CgEC124-C* using PCR method. The fusion fragment was amplified and introduced into *ΔCgEC124* mutant strains and tested using the vector pGTN primer.
