# Supplementary material for: Plant immunity suppression by an β-1,3-glucanase of the maize anthracnose pathogen Colletotrichum graminicola
Source: BMC Plant Biol. 2024 Apr 26;24:339. doi: 10.1186/s12870-024-05053-0 (PMC11046878; doi:10.1186/s12870-024-05053-0)
Supplement: Supplementary file 1 — Supplementary Material 1. [file 12870_2024_5053_MOESM1_ESM.zip › SUPPLEMENTARY FIGURE 3.pdf]

**SUPPLEMENTARY FIGURE 3**

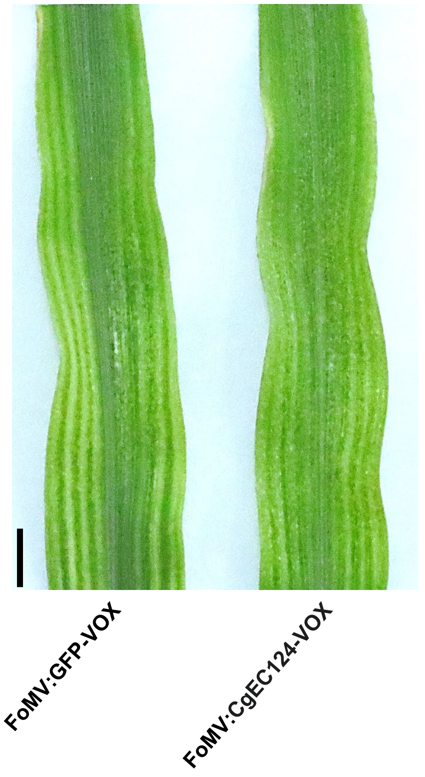


**SUPPLEMENTARY FIGURE 3 Phenotypes of FoMV:CgEC124-VOX maize plants.** Twelve-day-old plants were photographed. Scale bar=1 cm.
