# Supplementary material for: Plant immunity suppression by an β-1,3-glucanase of the maize anthracnose pathogen Colletotrichum graminicola
Source: BMC Plant Biol. 2024 Apr 26;24:339. doi: 10.1186/s12870-024-05053-0 (PMC11046878; doi:10.1186/s12870-024-05053-0)
Supplement: Supplementary file 1 — Supplementary Material 1. [file 12870_2024_5053_MOESM1_ESM.zip › SUPPLEMENTARY FIGURE 5.pdf]

**SUPPLEMENTARY FIGURE 5**

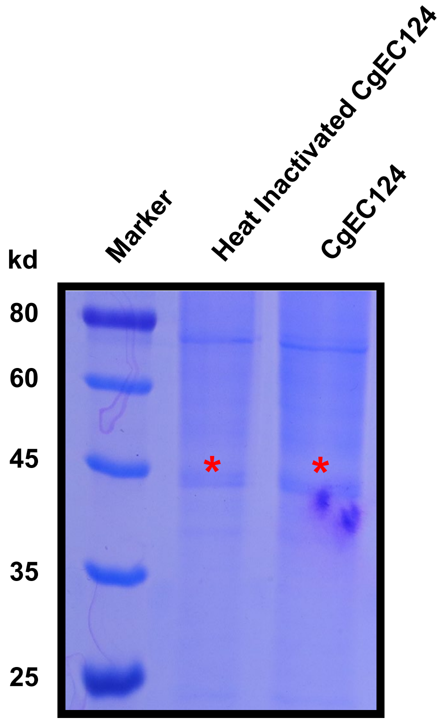


**SUPPLEMENTARY FIGURE 5 Purification of CgEC124 proteins from *Pichia pastoris*.** The purified His-tagged CgEC124 was stained by coomassie brilliant blue in SDS-PAGE gel.
