# Supplementary figures and images for: Plant immunity suppression by an β-1,3-glucanase of the maize anthracnose pathogen Colletotrichum graminicola
Source: BMC Plant Biol. 2024 Apr 26;24:339. doi: 10.1186/s12870-024-05053-0 (PMC11046878; doi:10.1186/s12870-024-05053-0)

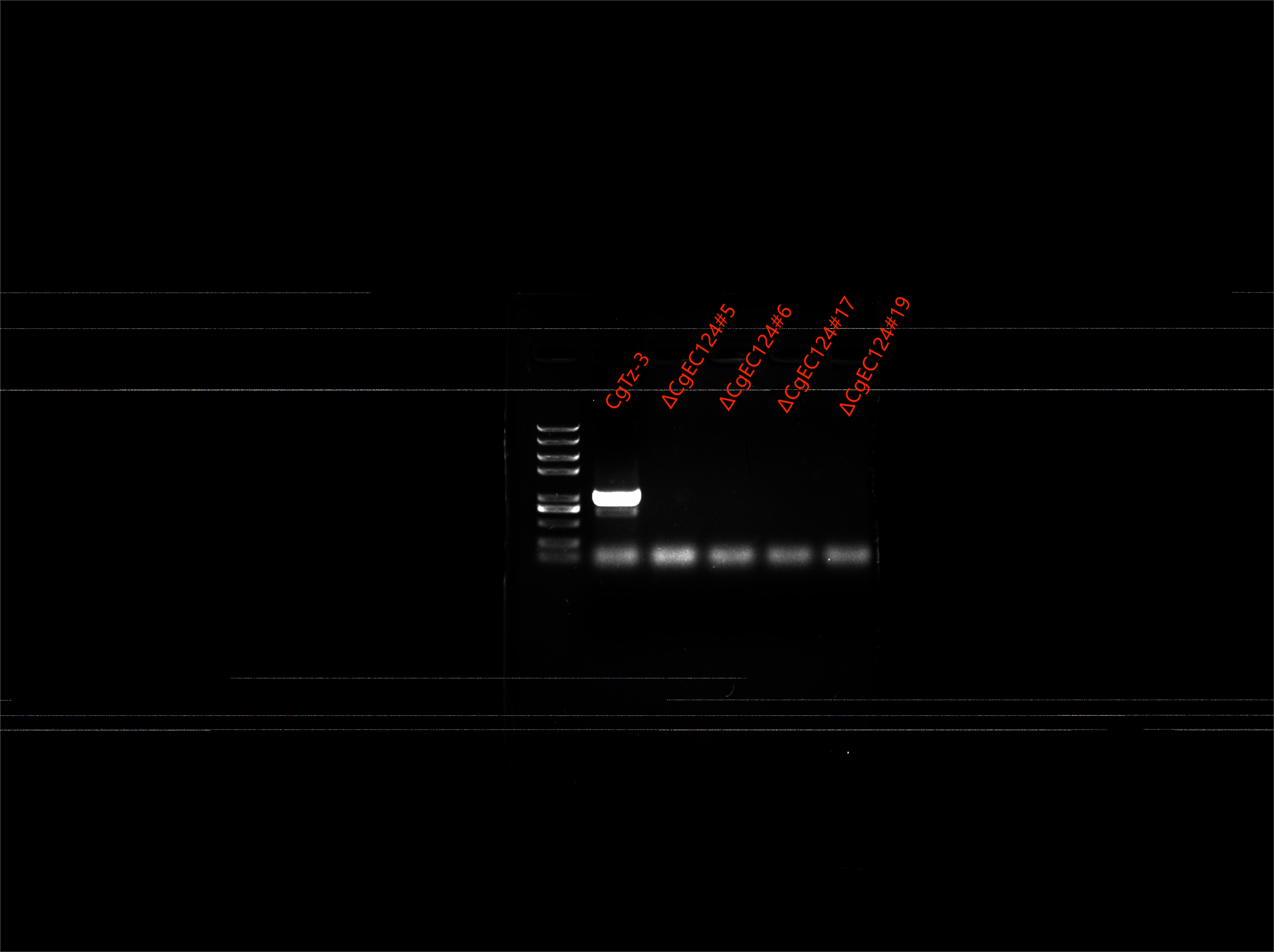

Supplement: Supplementary file 1 — Supplementary Material 1. [file 12870_2024_5053_MOESM1_ESM.zip › FIGURE S2A-1 uncropped gels.png]

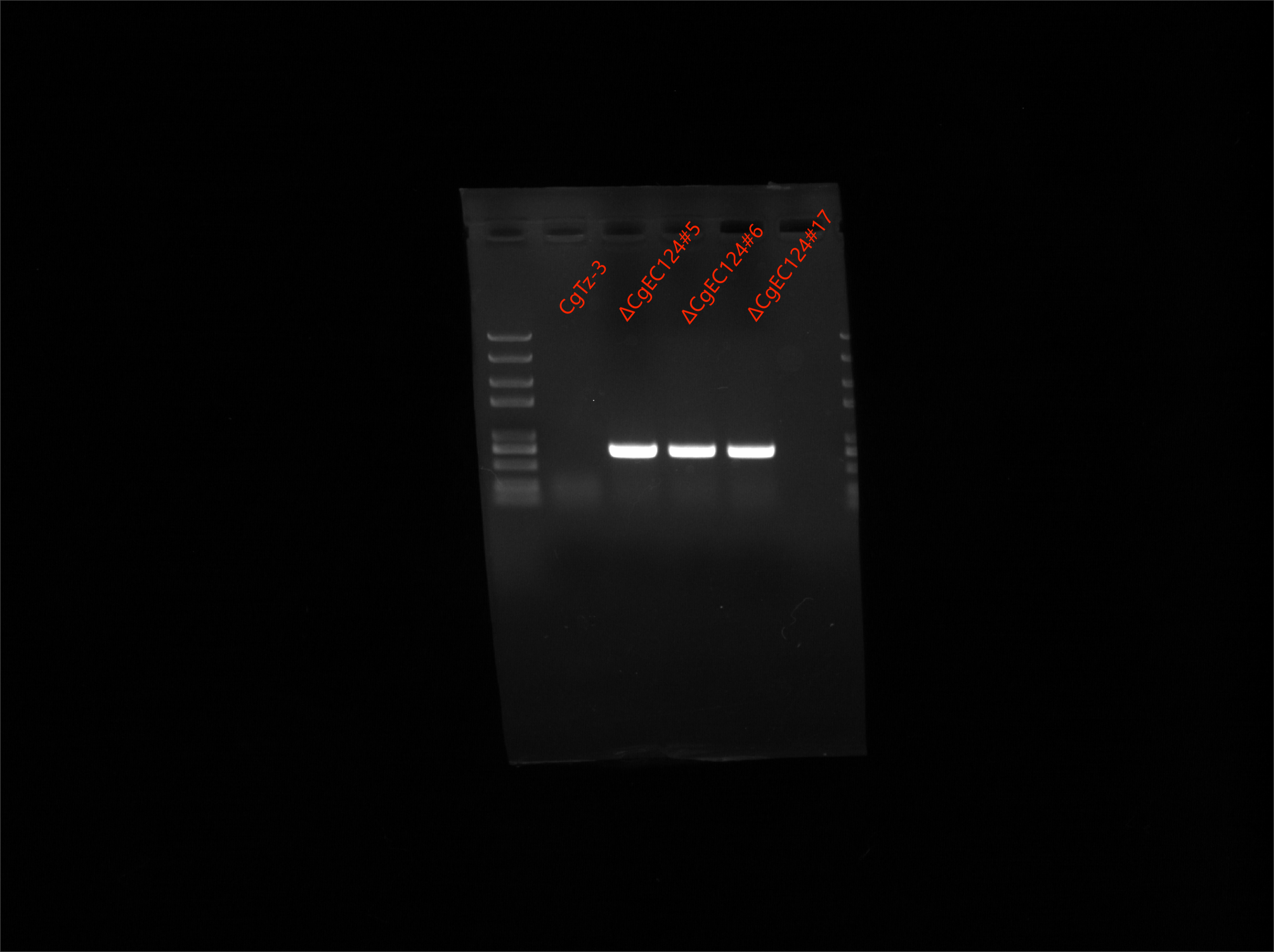

Supplement: Supplementary file 1 — Supplementary Material 1. [file 12870_2024_5053_MOESM1_ESM.zip › FIGURE S2A-2 uncropped gels.png]

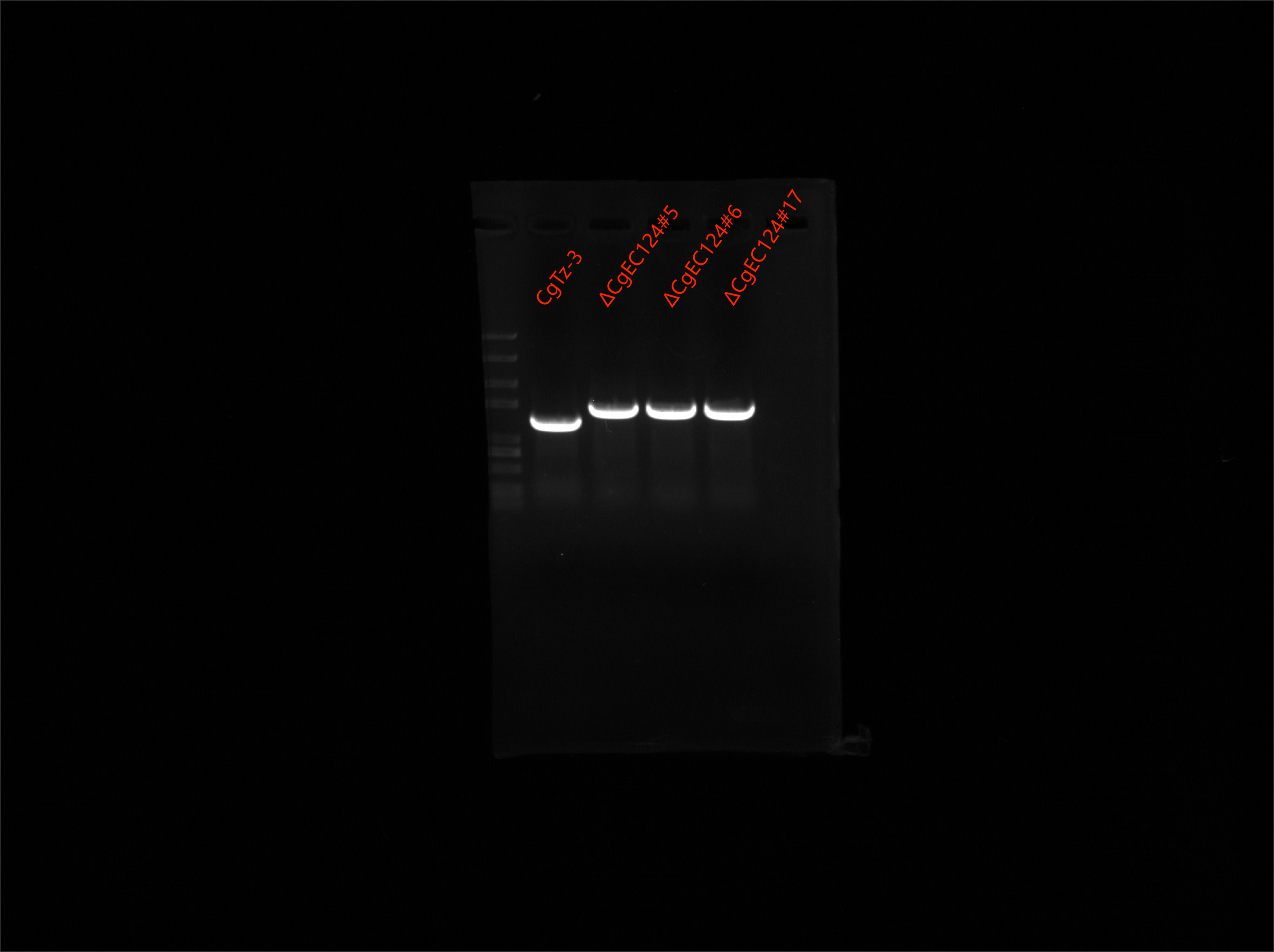

Supplement: Supplementary file 1 — Supplementary Material 1. [file 12870_2024_5053_MOESM1_ESM.zip › FIGURE S2A-3 uncropped gels.png]

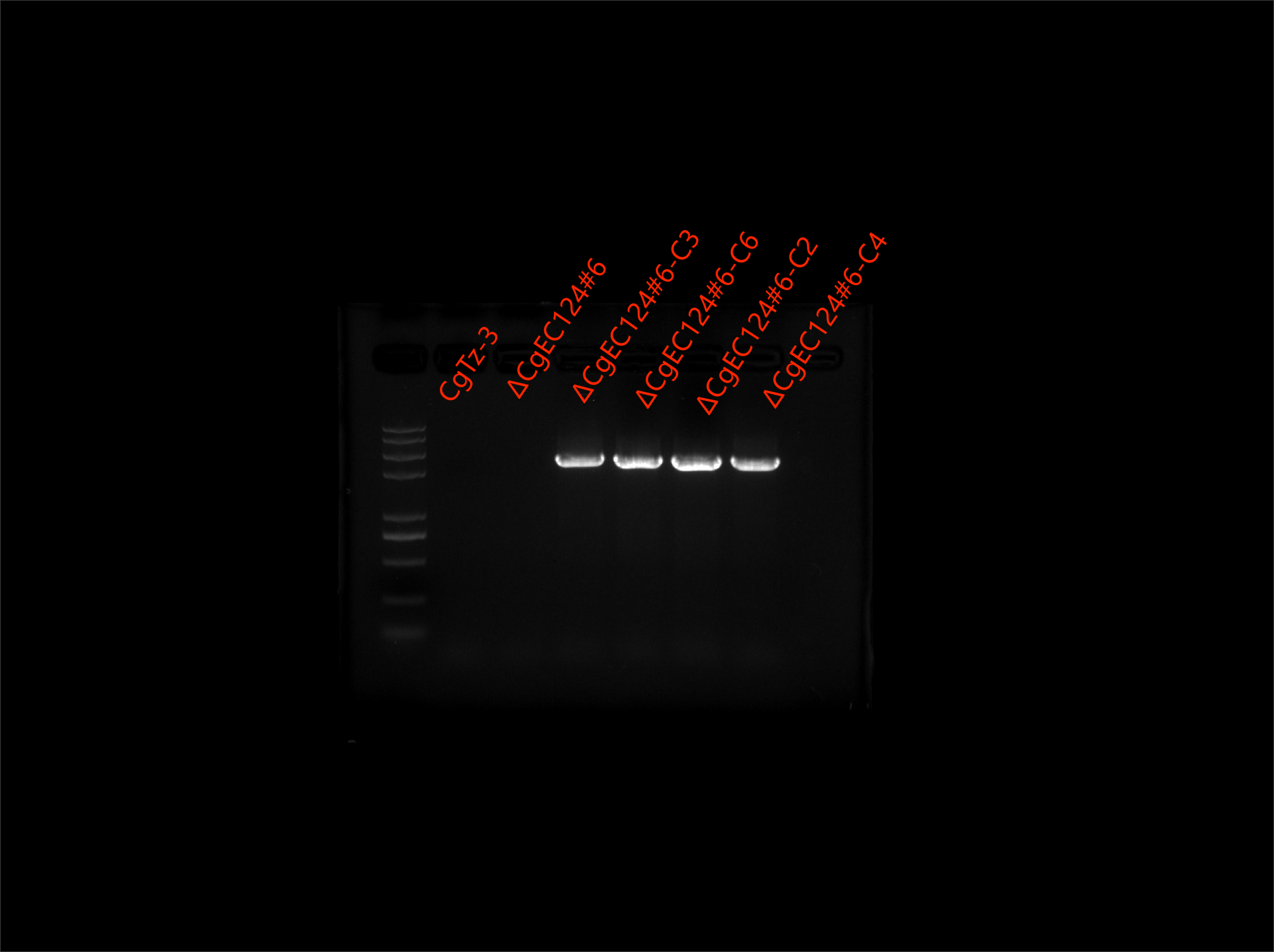

Supplement: Supplementary file 1 — Supplementary Material 1. [file 12870_2024_5053_MOESM1_ESM.zip › FIGURE S2B uncropped gels.png]

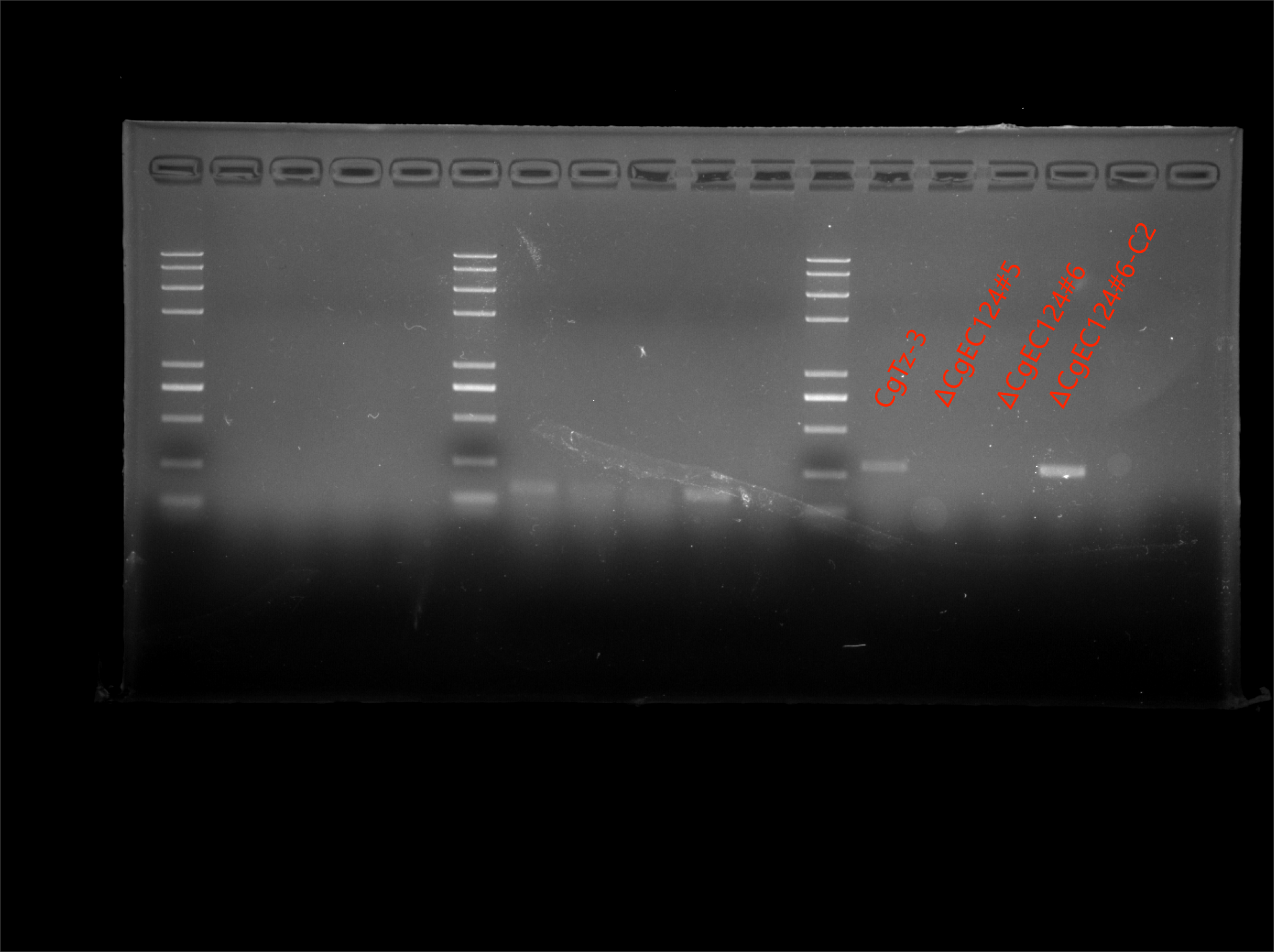

Supplement: Supplementary file 1 — Supplementary Material 1. [file 12870_2024_5053_MOESM1_ESM.zip › FIGURE S2C-1 uncropped gels.png]

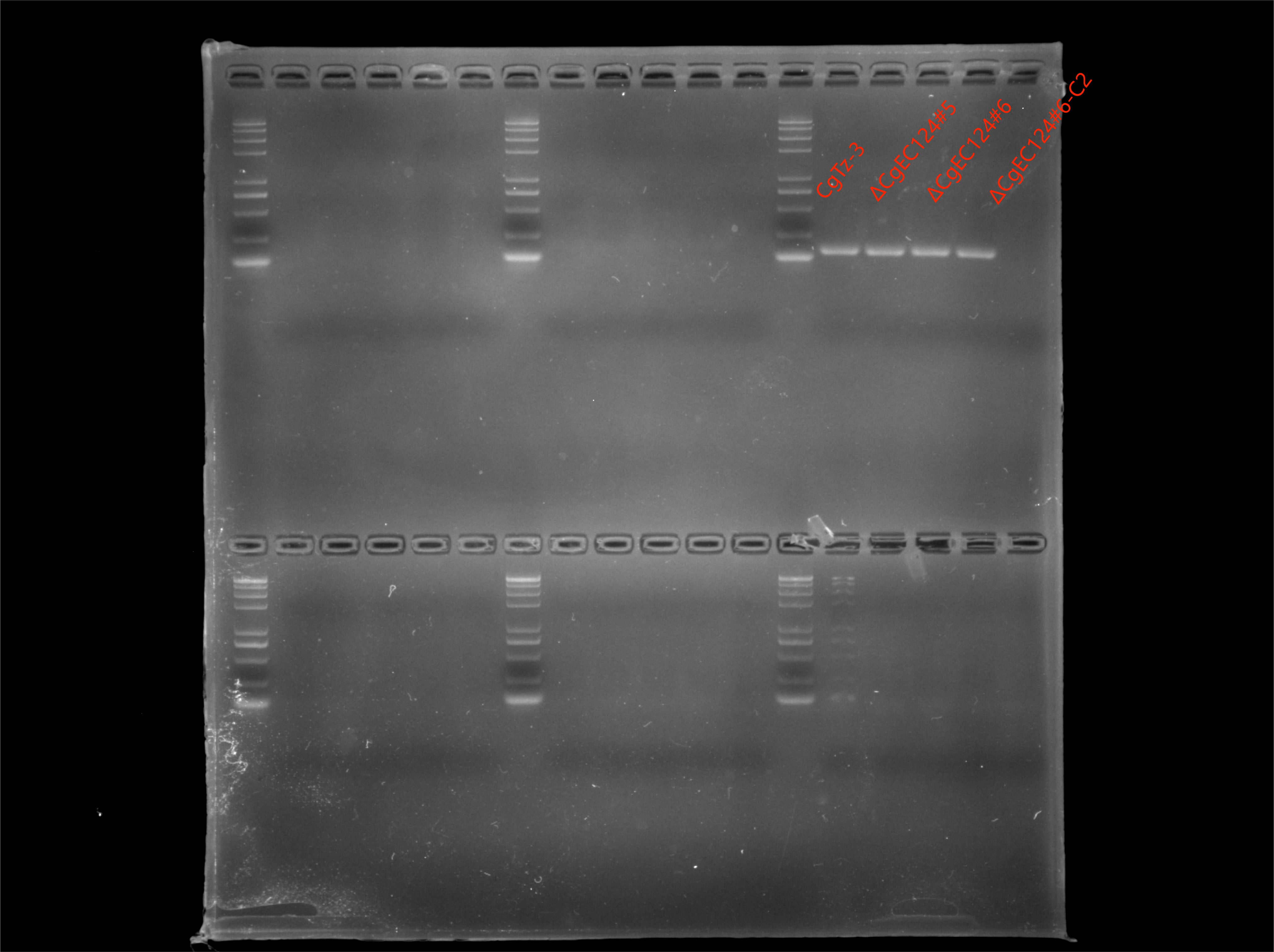

Supplement: Supplementary file 1 — Supplementary Material 1. [file 12870_2024_5053_MOESM1_ESM.zip › FIGURE S2C-2 uncropped gels.png]

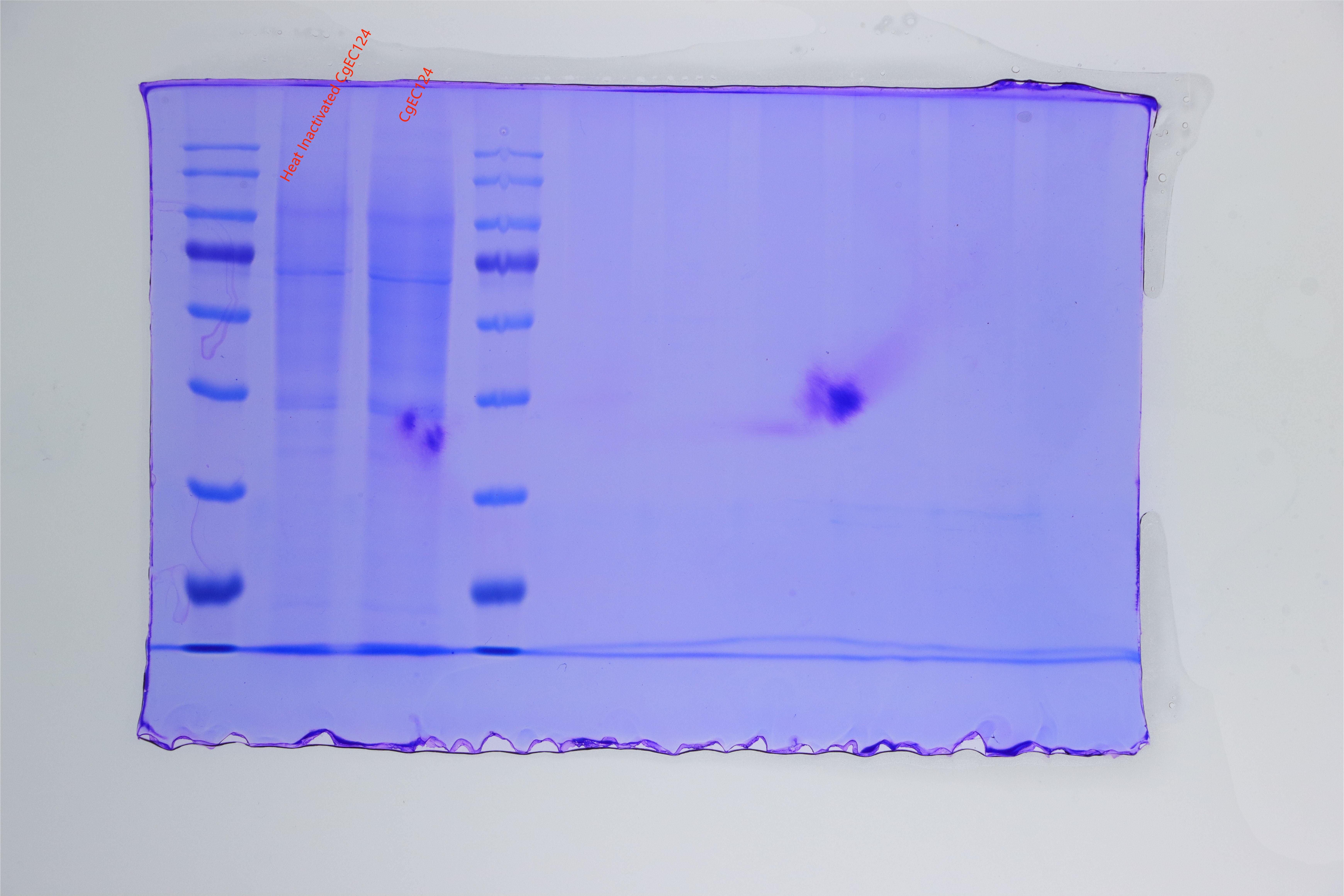

Supplement: Supplementary file 1 — Supplementary Material 1. [file 12870_2024_5053_MOESM1_ESM.zip › FIGURE S5 uncropped gels.JPG]
